# Drinking patterns and hydration biomarkers among young adults with different levels of habitual total drinking fluids intake in Baoding, Hebei Province, China: a cross-sectional study

**DOI:** 10.1186/s12889-020-08558-z

**Published:** 2020-04-08

**Authors:** Jianfen Zhang, Na Zhang, Yan Wang, Shuxin Liang, Shufang Liu, Songming Du, Yifan Xu, Hairong He, Hao Cai, Guansheng Ma

**Affiliations:** 1 Department of Nutrition and Food Hygiene, School of Public Health, Peking University, 38 Xue Yuan Road, Haidian District, Beijing, 100191 China; 2 Laboratory of Toxicological Research and Risk Assessment for Food Safety, Peking University, 38 Xue Yuan Road, Haidian District, Beijing, 100191 China; 3 Affiliated Hospital of Hebei University, 212 Yuhua Road, Lianchi District, Baoding, 071000 China; 4 School of Public Health, Hebei University Health Science Center, 342 Yuhua Road, Lianchi District, Baoding, 071000 China; 5 Chinese Nutrition Society, Room 1405, Beijing Broadcasting Building, No. 14 Jianguomenwai Street, Chaoyang District, Beijing, 100191 China

**Keywords:** Hydration biomarkers, Drinking patterns, Total drinking fluids

## Abstract

**Background:**

The purposes were to investigate the drinking patterns and hydration biomarkers among young adults with different levels of habitual total drinking fluids intake.

**Methods:**

A cross-sectional study was conducted among 159 young adults aged 18–23 years in Baoding, China. Total drinking fluids and water from food were assessed by 7-day 24-h fluid intake questionnaire and duplicate portion method, respectively. The osmolality and electrolyte concentrations of the 24 h urine and fasting blood samples were tested. Differences in LD_1_ (low drinker), LD_2_, LD_3_ and HD (high drinker) groups, stratified according to the quartiles of total drinking fluids, were compared using one-way ANOVA, Kruskal-Wallis H test and chi-square test.

**Results:**

A total of 156 participants (80 males and 76 females) completed the study. HD group had greater amounts of TWI (Total Water Intake), water from food, higher and lower contributions of total drinking fluids and water from food to TWI, respectively, than LD_1_, LD_2_ and LD_3_ groups (*p* < 0.05). Participants in HD group had higher amounts of water and water from dishes than participants in LD_1_, LD_2_ and LD_3_ groups (*p* < 0.05). No significant differences were found in the contributions of different fluids to total drinking fluids within the four groups (*p* > 0.05). The osmolality of urine was 59–143 mOsm/kg higher in LD_1_ than that in LD_2_, LD_3_ and HD group (*p* < 0.05). The percentage of participants in optimal hydration status increased from 12.8% in LD_1_ group to 56.4% in HD group (*p <* 0.05). HD and LD_3_ groups had 386~793 higher volumes of urine than that of LD_1_ and LD_2_ groups (*p* < 0.05). Differences were found in the concentrations of electrolytes among the four groups (*p* < 0.05). No significant differences were found in the plasma biomarkers (*p* > 0.05), with the exception of higher concentration of Mg in LD_3_ and HD groups than that in LD_1_ and LD_2_ groups (*p <* 0.05).

**Conclusions:**

Participants with higher total drinking fluids had better drinking pattern and hydration status. Interventions should be undertaken to advise adults to have adequate total drinking fluids, in order to keep in optimal hydration status.

**Trial registration:**

The registration number was ChiCTR-ROC-17010320, which was registered on the Chinese clinical trial registry.

## Background

Water is of vital importance for the health of humans. Water accounts for almost 50–60% of the total body mass of adults [1]. Moreover, water plays important role in facilitating cellular metabolism, modulating normal osmotic pressure and regulating the temperature of the body [2]. In addition, water intake has also been associated with mood state in women [3]. Insufficient water intake may lead body into dehydration. Researches have shown that dehydration can impair the physical [4, 5] and cognitive performances [6, 7], which included vigilance attention [8], working memory [9, 10] and executive function [11]. Moreover, dehydration may also have deleterious effects om cardiovascular health [12]. Therefore, it is important for people to be in optimal hydration status.

To keep the body functioning properly, people need to consume adequate volumes of water. Many factors such as individuals’ (age, gender, etc.) and environmental conditions (temperature, humidity, etc.) influence the requirements of fluids. Consequently, the dietary reference values of adequate intake of TWI (total water intake, including total drinking fluids and water from food), such as the EFSA (European Food Safety Authority) [13], the United States [14] and China [15] were different. Yet only 40% of men and 60% of women in 13 countries drank more than the adequate intake of water from fluids set by EFSA [16]. Among children from 19 countries, 60 ± 24% of them failed to have adequate water with guidelines, suggesting that children were not consuming enough water to be adequately hydrated [17]. Moreover, in China, about 32% of adults, children and adolescents drank less fluids than the recommendation [18], which potentially may have adverse effects on their health. Unfortunately, the hydration biomarkers were not measured in the studies mentioned above. Studies reporting the hydration status among people in free-living conditions were scarce. Data of 2009 to 2012 National Health and Nutrition Examination Survey demonstrated that 29.5% of US adults aged 20–74 years were not in optimal hydration status [19]. While among elderly aged ≥60 years from NHANES 2011–2014, 29.6% and 33.3% of women and men had Sosm (osmolality of serum) levels indicative of impending dehydration, with 10.2% and 12.6% of women and men had Sosm levels indicative of dehydration [20]. In China, there was merely one study exploring hydration status among male young adults, which showed that only 35.3% of them were in optimal hydration status [21]. Research showed that the participants with low total drinking fluids excreted significantly less volume of urine than those with high total drinking fluids over each 24 h period [22]. In addition, the osmolality, specific gravity of urine among adults with high total drinking fluids were lower than those with low total drinking fluids [22]. Nevertheless, the differences in hydration biomarkers among participants with different levels of habitual total drinking fluids intake were not explored yet in China. More studies should include hydration biomarkers to investigate the hydration status of Chinese adults in the future.

Drinking patterns, defined here as the amounts and types of fluids consumed, have been associated with health [23, 24]. Water, which could reduce the total energy intake, may contribute to the regulation of body weight and help individuals reach the recommendation of TWI [25–28]. Moreover, the replacement of diet beverages with water may improve the insulin resistance of people [28]. A rising number of studies asserted that there were positive associations between SSBs (sugar-sweetened beverages) and the risk of weight gain or obesity or metabolic syndrome or obesity-related cancers and type 2 diabetes in adults, children and adolescents [29–34]. Moreover, the consumption of SSBs was also associated with short sleep in middle school students [35]. Therefore, the sources of total drinking fluids were worth exploring. However, the drinking patterns may differ among people from different countries, for instance, the Chinese tend to have tea, while the Americans prefer to drink more coffee [36]. The drinking patterns were not only different from countries, differences also existed among people from the same country. In France, the volumes of fluids such as water, were higher among adults with higher total drinking fluids intake than those with lower total drinking fluids intake [22]. However, in China, there was no study investigated the drinking patterns among participants with different levels of habitual total drinking fluids.

In reference to the volumes of water from food, the contributions of water from food to TWI were different among countries, such as in the United States [37], in European countries [38] and in Japan [39]. Among adults in China, nearly 44% of the TWI came from food [40], however, the sources of water from food among young adults have not been reported. In addition, in France, the amount of water from food were 0.55 L and 0.68 L among participants with lower total drinking fluids intake and higher total drinking fluids intake, respectively [22]. However, there was no study investigating the differences in the sources of water from food among young adults with different levels of total drinking fluids. Therefore, it remains to be determined if participants with a low habitual total drinking fluids have a different contribution of water from food and different sources of water from food comparing with those with a high habitual total drinking fluids intake in Chinese.

The objectives of this study were, firstly, to investigate the differences in drinking patterns among young adults; secondly, to explore the hydration biomarkers among young adults with different levels of habitual total drinking fluids intake in free-living conditions. This will lead to the provision of a science-based education of fluids intake for young adults.

## Methods

### Participants

The inclusion criteria were that participants including males and females were healthy and aged 18–23 years. The exclusion criteria were that participants with smoking, habitual alcohol consumptions (> 20 g/day) or habitual high caffeine consumptions (> 250 mg/day) or had chronic diseases or other diseases (gastrointestinal tract disease, diabetes, oral disease, kidney disease, cardiovascular disease or other chronic or metabolic diseases) were excluded from the study [41].

### Sample size calculation

From a study performed among young male adults in China, the standard deviation of total drinking fluids was 468 mL [21]. Then, the SAS procedure (SAS Institute Inc., Cary, NC) was used to calculate the sample size. The δ set at 76 mL, α set at 0.05, and 10% drop-out rate was taken into account, 159 participants were needed in this study.

### Study procedure

**Fig. 1:**
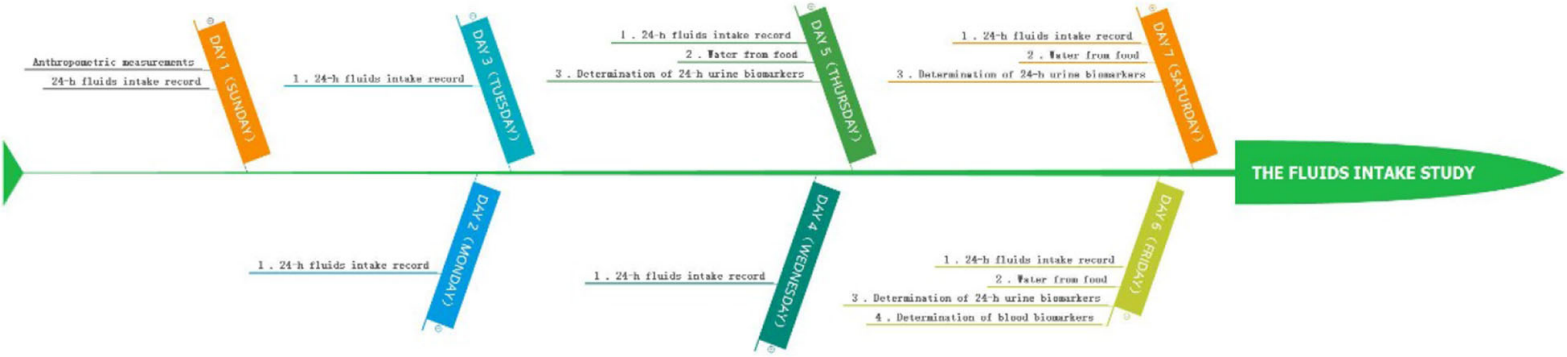
The study procedure

This was a cross-sectional study, which lasted for 7 consecutive days. On day 1, all participants were asked to take the anthropometric measurements including height, weight and waist circumference. After that, all participants were instructed to complete the self-designed 7-day 24-h fluid intake questionnaire to record the fluids intake from water and other beverages from day 1 to day 7. To ensure the competence and accurateness of the fluids intake, the questionnaires were checked by investigators every day. All the food the participants ate for three consecutive days (two weekdays and one weekend day, from day 5 to day 7) during the 7 consecutive days were weighed and recorded. From day 5 to day 7, 24-h urine samples including the first morning urine of three consecutive days (two weekdays and one weekend day) were collected by participants. On day 6, the fasting blood samples of all participants were collected. The temperature and humidity of indoor and outdoor was recorded each day for 7 days. The study procedure was shown in Fig. 1.

### Measurement of Total water intake

Total water intake (mL) = Total drinking fluids (mL) + Water from food (mL).

A self-designed 7-day 24-h fluid intake questionnaire was used to assess the total drinking fluids, as showed before. Moreover, the type and amount of drinking fluid for each time was measured using a cup to the nearest of 5 mL. T According to the General standard for beverages of China (GB/T 1–789-2015) [42], all the drinking fluids were classified as below: water (plain water, tap water and bottle water), tea (fermented tea and semi-fermented tea), milk and milk products (liquid milk, yogurt and other milk products), SSBs (carbonated drinks, sports drinks, sweetened fruit juice, vegetable juice, protein drinks and other sugared drinks), alcohols(wine, beer, liquor, etc.) and other beverages [43].

In order to assess the amount of water from food, all foods the participants ate before and after were weighed for three consecutive days of the 7 days (including two weekdays and one weekend day, from day 5 to day 7). The duplicate portion method, with samples of food being collected and sent to the laboratory immediately, was used to measure the water from food. Samples of all foods were measured according to the national standard of GB 5009.3–2016 [44] and the water from fruits or other snacks was assessed according to the China Food Composition Table (2009) [45]. The water from food was classified as below: staple food (steamed bread, steamed rice, etc.), dishes (vegetables, meat, fish and eggs), porridge (millet porridge and other porridges), soup (tomato egg soup and other soups) and snacks (fruits, nuts, etc.) [43].

### Temperature and humidity of the environment

The temperature and humidity of indoors and outdoors were recorded each day for 7 days (WSB-1-H2, Exasace, Zhengzhou, China).

### Anthropometric measurements

Height, weight and waist circumference were measured twice with the participants wearing light clothes and barefoot [15] (HDM-300; Huaju, Zhejiang, China; Accu Measure, Greenwood Village, CO, USA). [BMI: weight (kg) / height squared (m)].

### Urine biomarkers

The biomarkers of urine included the volumes, osmolality, pH, urine specific gravity (USG) and the concentrations of electrolyte.

24-h urine samples including the first morning urine were collected by participants using self-designed containers. Every urine sample was collected and tested immediately. Before being measured, all urine samples were stored at + 4°Cin refrigerator. Urine volume was measured to the nearest 0.1 g using a desktop electronic scale (YP20001, SPC, Shanghai, China). Urine osmolality was assessed with freezing point method by osmotic pressure molar concentration meter (SMC 30C; Tianhe, Tianjin, China). USG, pH, urea and creatinine were tested by automatic urinary sediment analyzer with uric dry-chemistry method (H-800; Dirui, Changchun, China). Urine electrolyte concentrations (including sodium, potassium, chloride, calcium, magnesium and phosphate) were measured by automatic biochemical analyzer with the ion-selective electrode potentiometer method (AU 5800; Beckman, Brea, CA, USA). The optimal hydration was defined when urine osmolality ≤500 mOsm/kg, middle hydration was defined as 500 mOsm/kg < urine osmolality ≤800 mOsm/kg, dehydration was defined as urine osmolality > 800 mOsm/kg [13, 46].

### Plasma biomarkers

Fasting blood samples were collected to measure the osmolality and electrolyte concentrations.

Plasma osmolality was assessed with freezing point method by osmotic pressure molar concentration meter (SMC 30C; Tianhe, Tianjin, China). The electrolyte concentrations (including sodium, potassium, chloride, calcium, magnesium and phosphate) were tested by automatic biochemical analyzer with the ion-selective electrode potentiometer method (AU 5800; Beckman, Brea, CA, USA).

### Statistics

The SAS 9.2 software (SAS Institute Inc., Cary, NC, USA) was used for statistical analysis. The results were reported as mean ± standard deviation (SD) if the data was normal distributed. While, median and quartile ranges (M and Q) was used to describe the data, which was not in normal distribution. Participants were divided into four groups, including LD_1_ (low drinker 1), LD_2_ (low drinker 2), LD_3_ (low drinker 3) and HD (high drinker) groups, according to the quartiles of total drinking fluids of participants (Q1: 324–858 mL, Q2: 859–1135 mL, Q3: 1136–1478 mL, Q4: 1479–3033 mL). One-way ANOVA, Kruskal-Wallis H test and chi-square test were used to compared the differences among the four groups. Differences between every two groups were compared using SNK (Student-Newman-Keuls) (*p* < 0.05). Significance levels were set at 0.05 (*p* < 0.05).

## Results

Eventually, a total of 159 participants were recruited, and 156 (80 males and 76 females) completed the study, with 98% complete rate. Table 1 showed the characteristics of the participants. Significant differences were found in the mean height and weight when comparing LD_2_, LD_3_, HD groups with group LD_1_, respectively (*p* < 0.05), with no differences between LD_2_, LD_3_ and HD groups. There were no significant differences in age and BMI among the four groups (*p* > 0.05).

**Table 1:** The characteristics of participants

	LD_1_ (*n* = 39)	LD_2_ (*n* = 39)	LD_3_ (*n* = 39)	HD (*n* = 39)	Total (*n* = 156)	*P*
Age (y)	19.6 ± 1.1	19.9 ± 1.0	19.8 ± 1.1	19.9 ± 1.0	19.8 ± 1.1	0.626
Height (cm)	162.6 ± 9.0^a^	168.0 ± 6.5	166.7 ± 8.6^b^	167.6 ± 8.1^c^	166.2 ± 8.3	0.014
Weight (kg)	56.3 ± 6.2^a^	63.5 ± 11.3	61.8 ± 13.6^b^	63.8 ± 12.5^c^	61.3 ± 11.5	0.013
BMI (kg/m^2^)	21.3 ± 2.0	22.4 ± 3.3	22.1 ± 4.1	22.6 ± 3.2	22.1 ± 3.3	0.333

Values are shown as the mean ± standard deviation (SD). BMI: Body Mass Index

^a^There was statistically significant difference between LD_1_ and LD_2_ groups, *p* < 0.05; ^b^There was statistically significant difference between LD_1_ and LD_3_ groups, *p* < 0.05; ^c^There was statistically significant difference between LD_1_ and HD groups, *p* < 0.05

### Temperature and humidity

The average indoor and outdoor temperature for the 7 days was 21.8 °C and 20.7 °C, respectively. The average indoor and outdoor humidity was 39.9% and 35.9%, respectively, see Table 2.

**Table 2:** The temperature and humidity of study days

	Indoors		Outdoors	
	Temperature (°C)	Humidity (%)	Temperature (°C)	Humidity (%)
Sunday	19.9	43	18.1	37
Monday	23.0	48	22.4	41
Tuesday	23.3	31	24.0	29
Wednesday	21.5	48	17.9	42
Thursday	21.5	40	21.0	36
Friday	22.2	35	19.2	35
Saturday	21.2	34	22.6	31

### Measurement of total water intake

TWI and water from food all increased with higher total drinking fluids among the four groups (*p* < 0.05). Participants in HD group had 515-1085 mL, 125–282 mL and 664–1383 mL more than those in LD_1_-LD_3_ groups in total drinking fluids, water from food and TWI (*p* < 0.05), respectively. Contributions of total drinking fluids to TWI were different among the four groups comparing with each other (*p* < 0.05), ranging from 38.7% in LD_1_ group to 59.1% in the HD group. The water from food accounted for 61.3–40.9% of TWI from LD_1_ group to HD group, which were also significantly different among the four groups (*p* < 0.05). Participants in HD group had a 20.4% higher and a 20.4% lower contribution of total drinking fluids and water from food to TWI than those in LD_1_ group, respectively (*p* < 0.05).

### Drinking patterns

The main contributor of total drinking fluids was water in the four groups, which accounted for 77.3–85.6% and there were no significant differences among LD_1_, LD_2_, LD_3_ and HD groups (*p* > 0.05). Significant differences were found in the amounts of water in the four groups (*p* < 0.05), with the amounts of water increased with higher total drinking fluids. Nevertheless, there were no statistically significantly differences in the amounts of tea, milk and milk products, SSBs, alcoholic and other beverages among the four groups (*p* > 0.05). Moreover, there were no significant differences in the contributions of tea, milk and milk products, SSBs, alcoholic and other beverages to total drinking fluids among the four groups (*p* > 0.05). SSBs were the second contributor to total drinking fluids followed water in the LD_1_, LD_2_, LD_3_ groups, but in the HD group, milk and milk products followed water.

### Water from food

Participants in HD group had 125–282 mL more water from food than those in other groups. The main contributor to water from food was dishes followed by staple food in all four groups. Participants in HD group had higher volumes of water from dishes and snacks than participants in LD_1_, LD_2_ and LD_3_ groups (*p* < 0.05). There were no statistically significant differences in the amounts of water from staple food, soup and porridge in the four groups (*p* > 0.05). Regarding the contributions of water from different foods, the contributions of water from snacks were different among the four groups (*p* < 0.05), as shown in Table 3.

**Table 3:** The TWI, total drinking fluids and water from food among participants consuming different levels of total drinking fluids

	LD_1_ (*n* = 39)			LD_2_ (*n* = 39)			LD_3_ (*n* = 39)			HD (*n* = 39)			Total (*n* = 156)		
	M	Q	%	M	Q	%	M	Q	%	M	Q	%	M	Q	%
Total drinking fluids (mL)	681^a^	226	38.7%^a^	967^d^	114	45.5%^d^	1251^b,f^	156	52.0%^b,f^	1766^c,e^	521	59.1%^c,e^	1135	620	50.6%
Water (mL)	564^a^	356	77.3%	745^d^	194	76.6%	1082^b,f^	351	79.5%	1613^c,e^	494	85.6%	866	642	81.0%
Tea (mL)	0	0	1.2%	0	0	0.8%	0	0	0.5%	0	0	1.2%	0	0	1.0%
Milk and milk products (mL)	39	71	7.1%	66	158	8.9%	21	146	6.6%	71	184	5.2%	43	131	6.6%
SSBs (mL)	36	106	10.3%	57	113	10.6%	46	173	9.2%	16	71	5.0%	43	112	8.0%
Alcohol (mL)	0	0	0%	0	0	0.4%	0	0	1.0%	0	0	0.8%	0	0	0.7%
Others (mL)	11	36	3.8%	0	21	2.5%	9	45	3.2%	0	40	2.0%	0	17	2.7%
Water from food (mL)	1018^a^	374	61.3%^a^	1134^b^	357	54.5%^d^	1175^b^	383	48.0%^b,f^	1300^c,e^	424	40.9%^c,e^	1174	373	49.4%
Staple food (mL)	274	145	26.4%	338	131	26.8%	297	152	26.3%	323	162	25.6%	301	141	26.3%
Dishes (mL)	544	228	52.5%	626	210	51.7%	624^b^	215	54.2%	676^c,e^	269	50.8%	620	217	52.2%
Soup (mL)	83	203	10.7%	34	194	8.6%	93	186	9.9%	104	202	11.2%	93	195	10.1%
Porridge (mL)	86	168	10.1%	132	202	12.1%	95	150	9.0%	92	259	10.5%	97	182	10.4%
Snacks (mL)	0	0	0.5%	0	0	0.8%	0^f^	0	0.6%^f^	0^c,e^	0	1.8%^c,e^	0	0	0.9%
Total water intake (mL)	1735^a^	467	_	2142^d^	467	_	2454^b,f^	339	_	3118^c,e^	709	_	2342	844	_

Values are shown as the median (M) and quartile ranges (Q)

^a^There was statistically significant difference between LD_1_ and LD_2_ groups, *p* < 0.05; ^b^There was statistically significant difference between LD_1_ and LD_3_ groups, *p* < 0.05; ^c^There was statistically significant difference between LD_1_ and HD groups, *p* < 0.05; ^d^There was statistically significant difference between LD_2_ and LD_3_ groups, *p* < 0.05; ^e^There was statistically significant difference between LD_2_ and HD groups, *p* < 0.05; ^f^There was statistically significant difference between LD_3_ and HD groups, *p* < 0.05

%: Contributions of total drinking fluids and water from food to TWI; percentages of different fluids in total drinking fluids; proportions of water from different foods in water from food

There were statistical significances in the amounts of TWI, total drinking fluids and water from food (*χ*^*2*^ = 121.262, *P*<0.001; *χ*^*2*^ = 145.319, *P*<0.001; *χ*^*2*^ = 18.941, *P*<0.001) among the four groups, respectively. There were statistical significances in the contributions of total drinking fluids and water from food to TWI, respectively (*F* = 86.910, *P* = 0.000; *F* = 86.910, *P* = 0.000). There were statistical significances in the consumption of water among the four groups (*χ*^*2*^ = 108.966, *P*<0.001). The water from dishes and snacks were different in the four groups (*χ*^*2*^ = 12.549, *P* = 0.006; *χ*^*2*^ = 8.746, *P* = 0.033), but no significant differences were found in the staple food, soup and porridge (*χ*^*2*^ = 7.377, *P* = 0.061; *χ*^*2*^ = 3.160, *P* = 0.368; *χ*^*2*^ = 1.838, *P* = 0.607). The contributions of snacks to water from food among the four groups were different (*χ*^*2*^ = 8.331, *P* = 0.040)

### Measurement of urine indexes

Table 4 showed that the volumes of urine increased from LD_1_ group, to HD group, while the osmolality, USG, and the concentrations of Na, K, Cl, Ca, Mg, phosphate, creatinine, uric acid and urea of urine were significantly different in LD_1_, LD_2_, LD_3_ and HD groups (*p* < 0.05). The percentages of participants in optimal hydration status was statistical higher in HD and LD_3_ groups than that in LD_1_, LD_2_ groups (*p* < 0.05).

**Table 4:** The characteristics of 24 h urine among participants consuming different levels of total drinking fluids

	LD_1_ (*n* = 39)		LD_2_ (*n* = 39)		LD_3_ (*n* = 39)		HD (*n* = 39)		Total (*n* = 156)	
	M	Q	M	Q	M	Q	M	Q	M	Q
24-h Volume (mL)	1076	370	1256^d^	500	1462^b,f^	671	1869^c,e^	747	1370	668
24-h urine Osmolality (mOsm/kg)	730	238	671	337	512	303	430^c,e^	280	587	309
(≤500 mOsm/kg, n, %)	5 (12.8%)^*^		13 (33.3%)		18 (46.2%)^b^		22 (56.4%)^c^		58 (37.2%)	
Na (mmol/L)	200	123	171	95	175	101	148^c^	110	175	101
K (mmol/L)	39.4	20	36.2	18.2	30.1^b,f^	19.7	26.7^c,e^	17.0	33.75	19.42
Cl (mmol/L)	202	106	174	101	168	114	148^c^	108	169	104
Mg (mmol/L)	2.90	0.96	3.11	1.39	2.42	1.26	1.89^c,e^	1.11	2.50	1.54
Ca (mmol/L)	2.47	2	2.21	1.59	2.10	1.25	1.55	1.06	2.09	1.54
Phosphate (mmol/L)	15.91	6.54	17.36	12.66	13.34	10.56	11.45^c,e^	9.05	14.89	8.91
Creatinine (mmol/L)	10.48	3.45	10.20^d^	6.82	8.24^b^	6.59	7.67^c^	4.97	8.95	5.17
Uric acid (mmol/L)	2.76	0.92	2.79	1.22	2.07^b^	1.66	1.96^c,e^	1.14	2.39	1.33
Urea (mmol/L)	230.30	108.13	231.50	162.87	179	135	154^c,e^	117	200.42	126.95
USG	1.015	0.003	1.017	0.007	1.015	0.006	1.013^c,e^	0.002	1.015	0.005
pH	6.8	0.5	6.7	0.3	6.7	0.3	6.7	0.3	6.7	0.3

Values are shown as the median (M) and quartile ranges (Q); ^*^χ^2^ = 23.215, *P* < 0.05;

^a^There was statistically significant difference between LD_1_ and LD_2_ groups, *p* < 0.05; ^b^There was statistically significant difference between LD_1_ and LD_3_ groups, *p* < 0.05; ^c^There was statistically significant difference between LD_1_ and HD groups, *p* < 0.05; ^d^There was statistically significant difference between LD_2_ and LD_3_ groups, *p* < 0.05; ^e^There was statistically significant difference between LD_2_ and HD groups, *p* < 0.05; ^f^There was statistically significant difference between LD_3_ and HD groups, *p* < 0.05

### Measurement of plasma indexes

Statistically significant differences were found in the concentration of Mg among the four groups (*p* < 0.05). No statistically significant differences were found in plasma osmolality, the concentrations of Na, K, Cl, Ca and phosphate among participants in the four groups (*p* > 0.05), as shown in Table 5.

**Table 5:** The characteristics of blood samples among participants consuming different levels of total drinking fluids

	LD_1_ (*n* = 39)				LD_2_ (*n* = 39)				LD_3_ (*n* = 39)				HD (*n* = 39)				Total (*n* = 156)				*P*
	M	Q	X	SD	M	Q	X	SD	M	Q	X	SD	M	Q	X	SD	M	Q	X	SD	
Osmolality (mOsm/kg)			299	5			298	6			300	5			299	5			299	5	0.519
Na (mmol/L)	140	1			141	2			141	2			141	2			141	2			0.095
K (mmol/L)	4.4	0.5			4.6	0.4			4.6	0.6			4.6	0.6			4.6	0.5			0.112
Cl (mmol/L)	104	2			104	3			104	2			104	2			104	2			0.660
Mg (mmol/L)	0.90	0.06			0.90^d^	0.05			0.92^b^	0.06			0.92^c,e^	0.08			0.91	0.07			0.013
Ca (mmol/L)			2.50	0.06			2.51	0.07			2.51	0.07			2.53	0.07			2.51	0.07	0.169
Phosphate (mmol/L)			1.32	0.13			1.31	0.16			1.31	0.17			1.28	0.18			1.30	0.16	0.676

Values are shown as the mean ± standard deviation (SD) and median (M) and quartile ranges (Q)

^a^There was statistically significant difference between LD_1_ and LD_2_ groups, *p* < 0.05; ^b^There was statistically significant difference between LD_1_ and LD_3_ groups, *p* < 0.05; ^c^There was statistically significant difference between LD_1_ and HD groups, *p* < 0.05; ^d^There was statistically significant difference between LD_2_ and LD_3_ groups, *p* < 0.05; ^e^There was statistically significant difference between LD_2_ and HD groups, *p* < 0.05; ^f^There was statistically significant difference between LD_3_ and HD groups, *p* < 0.05

## Discussion

In this study, when the total drinking fluids increased significantly, the water from food and TWI increased significantly accordingly among the four groups; the proportions of total drinking fluids in TWI were increased. Whereas, the contributions of water from food to TWI were decreased simultaneously, which were higher than the results among participants both in low and high drinkers in France [22]. The findings indicated that, participants with higher total drinking fluids may tend to prefer taking in more water from food. It meant that participants with lower total drinking fluids would not compensate from food to increase the intake of TWI. Therefore, they may have more danger to be in dehydration status than others. In addition, study performed among Europeans showed that, adults with higher TWI was associated with higher nutritional quality of the diet, assessed by several dietary indices [47]. Besides, it was also showed that high water intake remained significantly associated with higher die quality among children aged 2 to 18 years [48].

Researches demonstrated that, TWI was strongly associated with 24-h urine volume, osmolality, USG and solute concentrations [21, 49]. Moreover, urine osmolality ≤800 mOsm/kg [13, 46], was the indicator of optimal hydration status. In our study, when the total drinking fluids increased, the volume of 24-h urine increased, whereas, the 24-h osmolality, USG decreased among the four groups. Urine osmolality, USG and volume, but not plasma osmolality, responded to the changes in water intake [50]. Results of this study showed that, the 24-h urine osmolality of participants in LD_1_, LD_2_ and LD_3_ groups were higher than the standard, while the 24-h urine osmolality of participants in HD group was lower than the standard. Meanwhile, significantly more participants with the 24-h urine osmolality below the standard were in HD group than in other groups. Participants with lower total drinking fluids had less and more concentrated urine than participants with higher total drinking fluids, which indicated that participants with lower total drinking fluids maybe in dehydration, whereas, participants with high total drinking fluids maybe in optimal hydration status. Moreover, in France, the osmolality of urine among those with higher total drinking fluids and those with lower total drinking fluids were 371 mOsm/kg and 767 mOsm/kg, respectively [21, 22]. Blood homeostasis is well regulated by physiological compensations from the kidneys, even after 24 h of water intake deprivation [51]. Indeed, plasma osmolality was maintained at a wide range of water intakes from the study of NHANES ш [52]. Also, in our study, the plasma osmolality was 298–300 mOsm/kg with a wide range of total drinking fluids from 681 mL to 1766 mL, which indicated that plasma osmolality was not sensitive to the difference of total drinking fluids in free-living conditions, similarly to the study conducted before [14, 22, 53].

Regarding the drinking patterns, water was the main contributor of total drinking fluids among the four groups with the proportions higher than French, while the contributions of other beverages were lower than French [22]. The differences in culture and the accessibility of beverages could explain the observation. Regarding the types of beverages, SSBs have been shown to have detrimental effects on health [30, 54–56]. In our study, SSBs were the second contributor of total drinking fluids among participants in LD_1_, LD_2_ and LD_3_ groups, which contributed to over 10% of total drinking fluids, while the milk and milk products were the second contributor among participants in HD group. This observation suggested that participants with higher total drinking fluids may have healthier drinking behaviors. However, the consumptions of milk and milk products of participants in the four groups, which ranged from 21 mL to 71 mL, were much lower than the recommendation of China (300 mL) [57]. In China, 300 mL of milk was the main source of calcium, which could supply about 39% of the reference of the intake of calcium [15]. Therefore, more interventions should be undertaken to increase the consumptions of milk and milk products. In addition, a study demonstrated that drinking pattern based on water and milk among children was associated with better hydration including lower urine osmolality, while drinking regular soda and other drinks but not water was associated with inferior hydration [58].

In this study, water from food was also an important source of TWI, accounting for 40.9–61.3% to TWI among the four groups. Regarding the sources of water from food, dishes (such as vegetables, meat) and staple food (including steamed bread and rice) were the first and second contributor among the four groups. Moreover, participants with higher total drinking fluids had more amounts of water from dishes (such as vegetables) and snacks (for instance, the fruits) than those with lower total drinking fluids. But there were no significant differences in the contributions of water from different sources of food.

Research showed that heights were also different among the people of China and other countries due to race differences, which contributes to difference in body surface area and has corresponding effects on water requirements [59]. In this research, significant differences were found in the height and weight when comparing groups LD_2_, LD_3_, HD groups with LD_1_ group, respectively, but with no differences between LD_2_, LD_3_ and HD groups. However, the indexes, such as the amounts of total drinking fluids and water from food, were different between LD_2_, LD_3_ and HD groups. In addition, there were also significant differences of hydration biomarkers, for instance, the volume of urine between LD_2_, LD_3_ and HD groups. It meant that, the differences of anthropometric measurements at baseline did not influence the results of drinking patterns and hydration biomarkers among participants from different groups.

In terms of the strengths, it was the first study investigated the drinking patterns and hydration biomarkers among participants with different total drinking fluids. Moreover, 7-day 24 h fluid intake questionnaire was used to assess the total drinking fluids for seven consecutive days (two weekdays and one weekend day), which would reduce the recall bias. The duplicate portion method was used to assess the amounts of water from food, with all the foods were being weighed before and after participants ate for three consecutive days. In addition, 24 h urine was collected every time they urinated using specific container for three consecutive days (two weekdays and one weekend day), which would include incorporate factors fluctuating during weekdays and weekends. Moreover, the samples of urine collected by the participants, were stored at + 4 °C and sent to the laboratory for test immediately. All the tests were performed by professional investigators. Despite strengths mentioned above, the study employed did have limitations. This survey was performed with young adults only so extrapolation of the study findings to different age groups should be done with care. However, despite the limitation, the association between hydration status, drinking pattern and total drinking fluids intake remains meaningful. Future studies will determine the general application of our results to a wider population, including middle-aged adults and the elderly.

## Conclusions

Participants with higher total drinking fluids had higher water from food and TWI than those with lower total drinking fluids. Participants with higher total drinking fluids had better hydration status and drinking pattern than those with lower total drinking fluids. Interventions should be undertaken to encourage people to drink an adequate amount of total drinking fluids, preferably water, in order to be in optimal hydration.

## Data Availability

The datasets generated and/or analyzed during the current study are available from the corresponding author on reasonable request.

## Abbreviations

LD: Low drinker
HD: High drinker
TWI: Total water intake
EFSA: European Food Safety Authority
